# The Relationship between Canine Behavioral Disorders and Gut Microbiome and Future Therapeutic Perspectives

**DOI:** 10.3390/ani14142048

**Published:** 2024-07-12

**Authors:** Paula Kiełbik, Olga Witkowska-Piłaszewicz

**Affiliations:** Department of Large Animal Diseases and Clinic, Institute of Veterinary Medicine, Warsaw University of Life Sciences, 02-787 Warsaw, Poland

**Keywords:** canine behavioral disorders, gut–brain axis, fecal microbiota transplantation, gut dysbiosis, dog behavior

## Abstract

**Simple Summary:**

Canine behavioral disorders have become one of the most common concerns and challenging issues among dog owners. Therefore, to face this challenge, searching for novel therapeutic methods is highly required. Accumulated data show that mammals’ gut microbiome, immune system, and nervous system are in continuous communication and influence animal physiology and behavior. This review aimed to summarize and discuss the most important scientific pieces of evidence on the relationship between mental disorders and gut microbiota in dogs, simultaneously presenting comparable outcomes in humans and rodent models. A comprehensive overview of crucial mechanisms of the gut–brain axis is included. Additionally, the possible effects of the fecal microbiome transplantation procedure as a new tool to manipulate gut microbiota are discussed.

**Abstract:**

Canine behavioral disorders have become one of the most common concerns and challenging issues among dog owners. Thus, there is a great demand for knowledge about various factors affecting dogs’ emotions and well-being. Among them, the gut–brain axis seems to be particularly interesting, especially since in many instances the standard treatment or behavioral therapies insufficiently improve animal behavior. Therefore, to face this challenge, the search for novel therapeutic methods is highly required. Existing data show that mammals’ gut microbiome, immune system, and nervous system are in continuous communication and influence animal physiology and behavior. This review aimed to summarize and discuss the most important scientific evidence on the relationship between mental disorders and gut microbiota in dogs, simultaneously presenting comparable outcomes in humans and rodent models. A comprehensive overview of crucial mechanisms of the gut–brain axis is included. This refers especially to the neurotransmitters crucial for animal behavior, which are regulated by the gut microbiome, and to the main microbial metabolites—short-chain fatty acids (SCFAs). This review presents summarized data on gut dysbiosis in relation to the inflammation process within the organism, as well as the activation of the hypothalamic–pituitary–adrenal (HPA) axis. All of the above mechanisms are presented in this review in strict correlation with brain and/or behavioral changes in the animal. Additionally, according to human and laboratory animal studies, the gut microbiome appears to be altered in individuals with mental disorders; thus, various strategies to manipulate the gut microbiota are implemented. This refers also to the fecal microbiome transplantation (FMT) method, based on transferring the fecal matter from a donor into the gastrointestinal tract of a recipient in order to modulate the gut microbiota. In this review, the possible effects of the FMT procedure on animal behavioral disorders are discussed.

## 1. Introduction

Canine behavioral disorders have become one of the most common concerns and challenging issues among dog owners [1,2,3,4,5]. There is growing concern about dogs’ emotionality and welfare, especially since pets are treated as family members and play very important social roles in modern societies [6,7,8].

Interestingly, according to studies investigating pet owners’ experience during the COVID-19 pandemic, dogs became an important social and emotional support for their owners during this time [9,10]. Moreover, in one of the studies, the authors suggest that companion animals can help mitigate the effects of extreme stress and social isolation [11]. Consequently, nowadays, we can observe an increased effort to mitigate not only somatic but also mental issues in companion dogs.

The term “behavioral disorder” describes an animal behavior which appears to be undesirable or unexpected for the owner. Thus, lots of dog actions (classified as problematic for people) seem to be normal and natural activities for domestic canines. This refers to suspiciousness towards strangers, digging, chasing game, and barking, among others. This might be why many dog owners state that their dog expresses some behavioral problem (prevalence around 70–80%). Certainly, diagnosing dog behavioral abnormalities only based on owners’ subjective opinions can be misleading.

Nevertheless, even studies regarding the prevalence of canine behavioral problems reported by veterinarians or behaviorists show a high percentage of animals with behavioral issues [12,13,14]. In fact, canine undesirable behaviors can sorely impact dog–owner relationships, leading to animal relinquishment [14,15,16,17,18] or even euthanasia [19,20,21]. Moreover, recent studies have shown that pet behavioral problems might lead to poor mental health of the owners [22,23,24,25]. In one of the studies, which focused on exploring the experience of people owning problematic dogs, the authors concluded that all the examined owners experienced some level of frustration with their dog’s excitable behavior, and the majority of them were very frustrated [26]. Moreover, some canine behavioral disorders (aggressiveness towards people and/or animals) may become serious public health concerns. Especially dog bites are a worldwide public health concern, since they are the main risk factor for human rabies and affect mostly children [27,28,29]. However, it is worth mentioning that behavioral issues directly affect the welfare of the dog itself [30,31,32]. Stress, as an integral part of each dog’s behavioral disorder, seriously affects the organism, especially when it is prolonged. Impaired growth, reproduction, immune function, and reduced lifespan are examples of the many potential outcomes [33,34]. We can conclude that behavioral disorders in companion dogs may have serious consequences for the quality of life of both dogs and their owners.

## 2. Gut–Brain Connection

In many instances, standard treatment or behavioral therapies insufficiently improve animal behavior; thus, to face this challenge, searching for novel therapeutic methods is highly required [35,36]. The advanced approaches in this area should involve a detailed recognition of underlying physiological mechanisms, not only those widely recognized, such as neurological and hormonal changes or pain. Among the numerous factors contributing to canine behavioral disorders, the influence of gut-related mechanisms seems to be particularly interesting. This review aimed to summarize and discuss the most important scientific evidence on the relationship between mental disorders and gut microbiota in dogs, along with comparable outcomes observed in humans and rodent models. A comprehensive overview of the crucial mechanisms of the gut–brain axis is included in this review. These mechanisms include the homeostatic balance of the intestinal microbiome and crucial neurotransmitters and metabolites (mainly SCFAs) regulated by the gut microbiome, as well as the influence of the gut microbiome on inflammation and the hypothalamic–pituitary–adrenal (HPA) axis. All of the above mechanisms are presented in this review in strict correlation with brain and/or behavioral changes in the animal. Additionally, the possible effects of the manipulation of the gut microbiome by fecal microbiome transplantation on mental health are discussed.

### 2.1. Canine Gut Microbiome and Dysbiosis

The term microbiota refers to living microorganisms found in a defined environment, e.g., gut microbiota. These microbial communities are in symbiosis with the host, contributing to homeostasis and acting by regulating the organism’s physiological functions [37]. Within various segments of the canine gastrointestinal tract, different microbial communities can be found, but most bacterial sequences belong to the following phyla: Firmicutes, Fusobacteria, Bacteroidetes, Proteobacteria, and Actinobacteria [38]. The composition of gut microbiota differs among individuals, which is related to various diets, age, or geographical locations of the host. Interestingly, studies revealed some level of microbiota sharing between dogs and their owners [39,40]. Thus, the obtained results suggest that direct and frequent contact with our cohabitants may significantly shape the composition of our microbial communities. It is worth remembering that the term microbiome refers not only to microorganism composition but also to specific environmental conditions, microbial structural elements, and metabolites related to microbial activity. Thus, the microbiome creates a dynamic and interactive ecosystem that can change with time and interact with hosts [41].

The homeostatic balance of the intestinal microbiome is exceptionally beneficial to the host. A proper, healthy gut microbiome ensures a beneficial influence on the host’s immune system, defense against pathogens, or supply of vitamins and nutrients. Gut dysbiosis has been defined as a disturbance to gut microbiota homeostasis with further changes in their functional composition and metabolic activities [42,43]. Dysbiosis might have serious health consequences, as was proven in dogs as well. The gut microbiome is altered in many gastrointestinal diseases such as diarrhea, chronic enteropathies, and inflammatory bowel disease (IBD) but also in obesity, pancreatic insufficiency, or heart diseases in humans and dogs [44,45,46,47]. While the abovementioned diseases have been studied relatively extensively in relation to dysbiosis, there is still limited knowledge of how the gut microbiome influences dogs’ mental health, mood, and behavior.

### 2.2. Gut–Brain Axis

**Figure 1:**
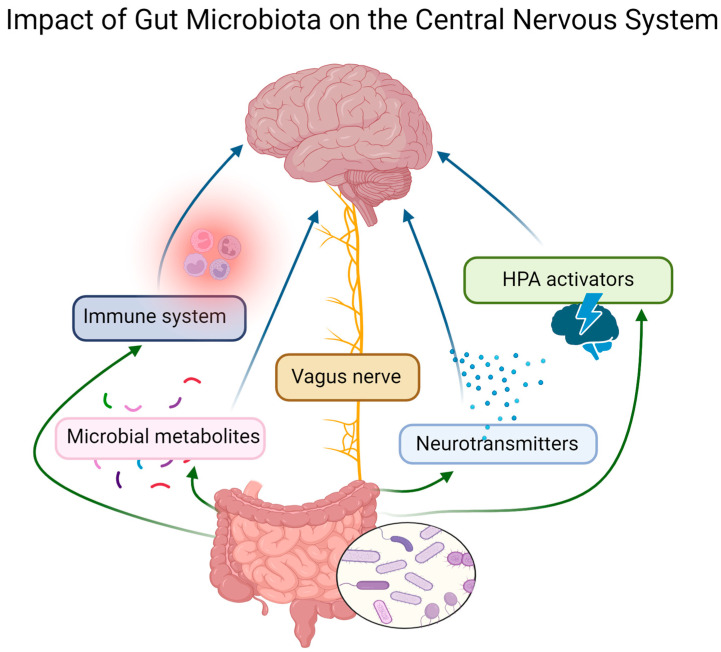
Crucial mechanisms of gut microbiome influence on the central nervous system. The gut microbiota has been found to influence the central nervous system through various mechanisms. This refers to the production/regulation of neurotransmitters, innervation via the vagus nerve, activation of the hypothalamic–pituitary–adrenal (HPA) axis, influence on the immune system, or production of microbiota metabolites.

The term gut–brain axis refers to the constant communication between the brain and gastrointestinal tract, and because of this, the enteric nervous system is often called the body’s “second brain”. This connection is bidirectional and affects various areas of animal life, including mood and behavior. The gut–brain axis is composed of immunological, metabolic, endocrinological, and neuronal mediators [48]. The microbiome can influence the animal central nervous system via the vagus nerve [49], neurotransmitter level regulation [50], the hypothalamic–pituitary–adrenal (HPA) axis [51], influence on the immune system [52], and production of metabolites [53] (Figure 1).

Human studies have shown that people with anxiety disorders and depression have more gastrointestinal symptoms (such as irritable bowel syndrome-like symptoms), compared to healthy individuals [54,55,56]. Similarly, altered microbiota composition was found to occur more often in other human mental diseases, including anxiety [57,58,59], schizophrenia [60,61], or posttraumatic stress disorder (PTSD) [62,63].

Similar studies as were conducted on humans have not been conducted as extensively on dog patients with behavioral disorders. The significance of this area of study seems to be high, especially since dogs show numerous physiological similarities to humans [64,65,66]. This refers also to the gastrointestinal tract. A recent study revealed that the gut microbiome of dogs is more similar to that of humans than that of mice and pigs, especially when lots of dogs eat the same food as their owners [67]. Interestingly, age-related differences were found in dog gut microbiome composition, pointing at a decreased diversity of the gut microbiome and reduced number of lactobacilli in older individuals [68,69]. These results suggest that the dog gut microbiome is likely to vary with age, as occurs in other animals, including humans. Additionally, dogs with better memory performance revealed a lower number of one of the genera of bacteria (*Actinobacteria*) in their fecal samples, which is in agreement with the high abundance of *Actinobacteria* in the gastrointestinal tract of persons living with Alzheimer’s disease [70]. Thus, the changes are similar to those reported in humans.

While most of the current literature focuses on humans, recent studies have shown differences in the composition of the gut microbiome between dogs with behavioral disorders and healthy individuals. In one of the studies, the gut microbiome structure and adrenocortical activity were investigated in dogs with aggressive and phobic behavioral disorders [71]. The obtained results revealed that aggressive behavioral disorder is characterized by a specific gut microbiome structure with a high biodiversity and enrichment in generally subdominant bacterial genera (*Catenibacterium* and *Megamonas* among others), compared to both phobic and normal behavior groups. On the other hand, phobic dogs were characterized by an enrichment in the *Lactobacillus* genus with well-known probiotic properties. The authors hypothesized that the dysbiotic microbiota of dogs with behavioral disorders (strictly related to long-term stress) can influence the local gut environment by releasing potentially neuroactive microbial by-products [71]. Similarly, another study revealed that the composition of the gut microbiome differs between aggressive and non-aggressive dogs [72]; however, the results are contradictory with those obtained by Mondo et al. [71]. Members of *Lactobacillus* bacteria were more abundant in the gut microbiomes of aggressive dogs [72], whereas in the previously cited study [71], the enrichment in the *Lactobacillus* genus was linked to phobic dogs. This inconsistency may be related to the methodology of canine behavioral disorder classification. Many aggressive behaviors are fear-based; thus, depending on the chosen criteria, animals can be classified as phobic (based on the underlying cause of the unwanted behavior) or aggressive (based on the observable, unwanted behavior) dogs. Nevertheless, further study on the canine gut microbiome role may certainly help clarify whether or how this can influence canine aggression. Kirchoff et al. also reported higher *Proteobacteria* and *Fusobacteria* abundances in non-aggressive dogs, whereas *Firmicutes* were more abundant in aggressive animals [72]. Craddock et al. characterized the microbiota of working dogs and determined if the composition of the microbiota is associated with behavioral and performance outcomes [73]. The obtained results showed an increased abundance of *Firmicutes* in aggressive dogs, which is in line with findings from Kichoff et al. [72]. The authors also observed increased *Lactobacillus* in association with phobic behavior, as well as the increased richness of gut microbiota among more aggressive individuals, which is consistent with the study of Mondo et al. [71]. Craddock et al. [73] also identified increased *Ruminococcus* abundance in association with increased canine aggression, while Mondo et al. [71] linked this member of the canine gut microbiota with phobic behavior. Other results obtained by Pellowe et al. [74] revealed an increased richness of gut microbiota in both aggressive and anxiety groups of dogs, similar to results obtained by Mondo et al. [71] and Craddock et al. [73]. Studies performed by Pellowe et al. also suggest a strong relationship between the genus *Blautia* and anxiety in domestic dogs [74]. Interestingly, in dogs, a significantly decreased level of genus *Blautia* within intestinal microbiota was observed in dogs with gastrointestinal disease, especially acute hemorrhagic diarrhea [75].

Studies cited in the following review suggest that there is an urgent need to deepen the knowledge on the mechanisms underlying the relationship between canine behavioral disorders and the altered composition of the gut microbiome. Future investigations in this area of study should also consider the individual dog core microbial population in the gut. The biodiversity of the canine gut microbiome may naturally occur in various diet compositions or geographical locations.

### 2.3. Gut Microbiome and Neurotransmitters

Gut microbiota can influence brain function by regulating crucial animal behavior neurotransmitters such as serotonin (5-HT), gamma-aminobutyric acid (GABA), acetylcholine, dopamine, or norepinephrine [76]. The imbalance of neurotransmitters is one of the reasons responsible for distress and mental disorders; thus, we can hypothesize that the gut microbiome influences mental health, mood, and behavior. Summarized information regarding serotonin, GABA, and dopamine in relation to animal behavior is presented in Table 1.

Serotonin plays a crucial role in animal behavior by regulating an animal’s mood, sleep, cognition, social interactions, and anxiety [77]. In general, serotonin is known as the “happiness hormone” produced due to the transformation of tryptophan. According to studies, more than 90% of total body serotonin is produced in the gut by specialized endocrine cells enterochromaffin cells (ECs), mucosal mast cells, and neurons from the enteric nervous system [78]. Serotonin is an important factor acting locally in the gastrointestinal tract by influencing intestinal peristalsis, motility, secretion, vasodilatation, and the absorption of nutrients. The exact mechanism explaining how peripheral serotonin could influence brain functionality is not clear; however, the link between the systemic serotonin system and animal behavior has been recognized [79,80]. The importance of the gut microbiome’s role in regulating blood serotonin levels was proven. In germ-free mice (an animal model without microbial colonization), a significant decrease in serum serotonin level was detected [81]. In another study in mice, following 4 weeks of antibiotic treatments, the richness and diversity of intestinal microbiota and serotonin levels decreased significantly [82]. Additionally, in one of the studies, the authors reported reduced levels of serotonin in patients with gut microbiome-related dysbiosis—irritable bowel syndrome (IBS)—compared to healthy controls. Interestingly, this study also revealed a correlation between decreased levels of serotonin and psychological state changes in tested patients [83]. Likewise, a 50% reduction in serotonin in intestine mucosa was found in mice mimicking autism syndrome [84], which is consistent with another study where reduced intestinal serotonin synthesis was found in children with autism spectrum disorder [85]. Therefore, study evidence (mostly based on mice and humans) shows that mental health disorders may be related with the disturbance in gut-related serotonin metabolism.

In the case of canine behavioral disorders, several studies have reported a relationship between a decreased level of serotonin and undesirable behaviors. Researchers detected significantly lower serum serotonin concentrations in aggressive dogs, compared to non-aggressive individuals [86,87,88]. To the best of the authors’ knowledge, no study has been conducted to investigate the relationship between canine behavioral disorders and the disturbance in gut-related serotonin metabolism. In one of the studies, the effect of a novel nutraceutical supplement (containing 5-HTP—the intermediate metabolite of L-tryptophan in the biosynthesis of serotonin) on the fecal microbiome and stress-related behaviors in dogs was investigated. Serum serotonin levels were not measured in this study; however, supplementation revealed an improvement in both gastrointestinal disturbances (vomiting episodes and diarrhea) and behavioral disorders (aggressiveness, nervousness, alertness, hiding and isolating, fearfulness) [89].

Another example of a neurotransmitter regulated by gut microbial function is dopamine. This catecholamine regulates crucial central and peripheral nervous system functions, including reward and motivation [90,91,92,93]. Dopamine is a vital neurotransmitter for mental disorders including depression, which was previously associated only with noradrenergic and serotonergic system dysfunction. Current studies have proven the role of dopaminergic dysfunction in the pathophysiology of major depression [94,95]. It is of great importance that in humans more than 50% of dopamine is synthesized in the gastrointestinal tract and the overall dopamine level is influenced by gut microbiota [96]. In germ-free mice, the level of free dopamine was decreased compared to specific pathogen-free mice. Germ-free mice also exhibited an increased turnover rate of dopamine in the brain [97]. The obtained results indicate that the gut microbiota plays a crucial role in the production of free catecholamines (including dopamine) in the gut lumen. Significant evidence supports the involvement of some key microbial genera in dopamine production, release, and bioavailability. Microbiota dysbiosis can lead to dopaminergic deficits that are related to pathological conditions such as Parkinson’s disease [98]. In dogs, lower levels of the urinary dopamine/serotonin ratio have been associated with impulsivity in dogs [99], whereas increased levels of plasma dopamine and serotonin have been detected in anxious dogs [100]. In one study, dogs with ADHD-like behaviors showed lower serotonin and dopamine serum concentrations [101]. Unfortunately, none of the cited studies performed an analysis of the gut microbiota and its potential involvement in dopamine system dysregulation in correlation with dog behavioral disorders.

Gamma-aminobutyric acid (GABA) is a main inhibitory neurotransmitter in the central nervous system with important physiological and behavioral functions such as the regulation of mood, anxiety, sleep, or memory enhancement [102,103,104]. GABAergic neurotransmission inhibits the amygdala and prevents inappropriate emotional and behavioral responses [105]. Reduced GABA plasma concentrations and GABA concentrations in prefrontal brain regions have been reported in anxiety states, stress-related disorders, and depression in humans [106,107,108]. A broad diversity of bacteria has been reported to produce/influence GABA in human gut microbiota, and reports suggest that the manipulation of the gut microbiota may impact GABA levels [109]. Moreover, researchers found that oral GABA administration in mice could elevate the production of total SCFAs (short-chain fatty acids), which play a crucial role in intestinal tract health [110]. Promising outcomes in this area of study led to increased research studies into the development of food products containing GABA for calming effects [111,112,113]. The alleviative effects of administered GABA were also evaluated on behavioral abnormalities in aged dogs. The obtained results revealed an improvement in emotional states, with no adverse effects [114]. Similarly, calming effects were observed in another study, where orally administered GABA reduced activity and urinary cortisol levels in the examined dogs [115]. It seems that GABA can also be influenced by diet in dogs. In one study, dogs on a BARF diet (Feeding Bones and Raw Food) revealed higher levels of GABA in their feces, as well as a different microbial composition (significantly higher abundance of *Escherichia coli* and *Clostridium*), compared to commercially fed dogs [116].

**Table 1:** Representative behavior-related neurotransmitters regulated by the gut microbiota. The table presents the probable role of the neurotransmitter in animal behavior, data on the role of the gut microbiota in its regulation, and canine studies in the area of behavioral disorders in connection with the neurotransmitter.

Neurotransmitter	Effect on Animal Behavior	Gut Microbiota Regulation	Canine Behavioral Disorder
Serotonin	Regulates mood, sleep, cognition, social interactions, and anxiety [77]	Germ-free mice revealed a significant decrease in serum serotonin level [81] Significant decrease in serum serotonin level after 4 weeks of antibiotic treatments in mice [82] Reduced serotonin serum level in human patients with IBS [83] Reduced intestinal serotonin synthesis in children with autism spectrum disorder [85]	Significantly lower serotonin serum level in aggressive dogs [86,87,88]
Dopamine	Regulates reward-related behavior and motivation [90,91,92,93]	Germ-free mice revealed decreased level of free dopamine and increased turnover rate of dopamine in the brain [97] Microbiota dysbiosis can potentially lead to dopaminergic deficits (related with Parkinson’s disease, among others) [98]	Lower level of urinary dopamine/serotonin associated with impulsivity in dogs [99] Increased level of plasma dopamine in anxious dogs [100] and dogs with ADHD-like behaviors [101]
Gamma-aminobutyric acid (GABA)	Regulates mood and anxiety and prevents inappropriate emotional and behavioral responses [102,103,104,105]	Various bacteria produce/influence GABA in human gut microbiota, and manipulation of gut microbiota may impact GABA levels [109]	Reduced activity and urinary cortisol level in dogs after orally administered GABA [115]

### 2.4. Main Microbial Metabolites—Short-Chain Fatty Acids (SCFAs)

Gut microbiota produces metabolites such as short-chain fatty acids (SCFAs), which seem to play a key role in intestinal and overall homeostasis. Acetate, propionate, and butyrate are three major SCFAs derived from the intestinal microbial fermentation of undigested dietary fibers [117,118,119]. Moreover, since these undigested polysaccharides are fermented by gut microbiota, the analysis of the SCFA levels can directly help to assess the gut microbiota composition. SCFAs act as an energy source for colonic epithelial cells with numerous health benefits, including anti-inflammatory, immunoregulatory, anti-obesity, anti-diabetes, anticancer, cardiovascular protective, and hepatoprotective activity [120]. It is also widely accepted that microbial dysbiosis can lead to the altered production of microbial metabolites, including decreased SCFA levels [121]. This refers to dogs, as well. Acute diarrhea leads to dysbiosis with significant alteration in fecal SCFA profiles, among others. The abundance of SCFA-producing bacteria was reduced in fecal samples of dogs with acute diarrhea [122], chronic enteropathies [123], or inflammatory bowel diseases (IBDs) [124].

It is worth mentioning that SCFAs also play a crucial role in the communication between the brain and gastrointestinal tract, however, the underlying mechanisms through which SCFAs influence the brain and behavior have not been fully clarified. These compounds are involved in maintaining integrity of the intestinal barrier and preventing the translocation of bacterial products, which can lead to increased production of cytokines and affect the blood–brain barrier (BBB) [125]. Likewise, SCFAs regulate microglia functions and BBB integrity. Increased permeability of BBB is related to various neurological disorders such as neuroinflammation or neurodegeneration [126]. Impaired integrity of BBB, caused as a consequence of altered SCFA concentrations, can lead to the hypothalamic–pituitary–adrenal (HPA) axis activation or systemic inflammation and indirectly affect animal mood and behavior [127].

Studies have shown that changed gut microbiota with altered SCFAs production is related to mental and neurologic pathologies, including Parkinson’s disease [128], Alzheimer’s disease [129], and autism spectrum disorder [130]. The potential interactions between the abundance of SCFA-producing bacteria and behavioral pathologies were also considered. In one study, the authors concluded that gastrointestinal illnesses or any disruptions related to the gut microbiome (such as IBD) are often worsened during stressful periods [131]. Similarly, an association between fecal SCFA levels and depressive symptoms among women was indicated [132]. A study on the non-human primate model of depression also revealed that peripheral (serum) and central (cerebrospinal fluid) SCFAs are implicated in the onset of depression [133]. An interesting experiment was recently performed on mice with a depleted microbiome. The authors of the study revealed that orally administered SCFAs decreased anxiety-like behavior in the tested mice [134]. Similar studies were not conducted on dogs; however, a deeper understanding of the interaction between SCFA levels and canine behavioral disorders is exceedingly required.

### 2.5. Gut Dysbiosis and Inflammation

A healthy microbiome protects the body against excessive inflammatory reactions, simultaneously inducing intestinal immune responses during the invasion of pathogens. Thus, we can conclude that a properly functioning microbiome may have both pro- and anti-inflammatory effects, depending on the situation [135]. Changes in the gut microbial composition and/or overall organism homeostatic imbalance result in a pro-inflammatory state induced by gut microbiota. In response, the body produces effector molecules (cytokines and other mediators) to initiate an inflammatory response [136,137,138]. Chronic inflammation and gut dysbiosis underlay many chronic multisystem conditions in dogs, including chronic inflammatory enteropathy [139,140], IBD [141,142], cardiovascular diseases [143,144], and arthritis [145]. Increasing evidence also indicates the role of ongoing inflammation in behavioral disorders. Elevated levels of pro-inflammatory cytokines (including Tumor Necrosis Factor-alpha (TNF-alpha) and Interleukin 6 (IL-6)) influence brain function, leading to depression, anxiety, and anger in humans [146,147,148]. The addition of probiotics to the standard medications used for mental disorder treatment can decrease the level of pro-inflammatory cytokines, as was proven in human patients [149] and mice [150] suffering from chronic inflammation. In one study, the inhibition of pro-inflammatory cytokines was achieved by introducing Lactobacillus mucosae NK41 and Bifidobacterium longum NK46 to mice with induced anxiety-like/depressive behaviors [151]. The obtained results revealed that the administered gut bacteria can alleviate anxiety/depression and colitis by suppressing gut dysbiosis.

The human and mouse studies indicate that depression and anxiety disorders are associated with chronic inflammation and gut dysbiosis. In the case of canine behavioral disorders, no similar studies were performed. Inflammatory processes have been widely studied in relation to diet and its possible anti-inflammatory effect [152,153,154]. In one study, the authors tested the hypothesis that an elevation in inflammatory markers (C-reactive protein, IL-6) could be associated with the presence of aggressive behavior in dogs. The obtained results showed higher levels of inflammatory markers in dogs with aggression, compared to non-aggressive individuals [155]. However, possible interactions between gut dysbiosis, inflammation, and behavior were not explored in dogs.

### 2.6. Gut Dysbiosis and Hypothalamic–Pituitary–Adrenal (HPA) Axis

Another important mechanism involved in the crosstalk between the gut microbiota and brain is through the modulation of the hypothalamic–pituitary–adrenal (HPA) axis. The HPA axis is the main physiological system that modulates a wide variety of behavioral processes, especially body stress response, but also rewarding behaviors, learning, and memory [156,157,158]. Stressful situations lead to the activation of the HPA axis, invoking corticotrophin-releasing hormone (CRH) release from the hypothalamus and the secretion of adrenocorticotropic hormone (ACTH). Circulating ACTH stimulates glucocorticoid hormone synthesis and secretion from the adrenal glands [159,160]. Since the activation of the HPA axis is essential for survival during stressful situations, the chronic elevation of stress hormones can lead to multiple organ systems’ dysregulation and has clinical consequences [161].

**Figure 2:**
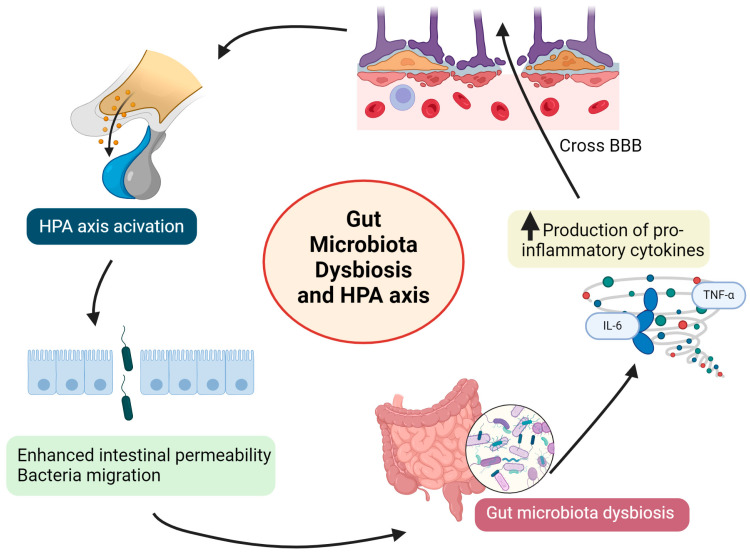
Modulation of the hypothalamic–pituitary–adrenal (HPA) axis by gut microbiota dysbiosis. The figure shows one of the proposed mechanisms linking gut microbiota dysbiosis and the HPA axis. Gut dysbiosis may contribute to the enhanced production of pro-inflammatory cytokines, and some of these molecules (including TNF-alpha and IL-6) might cross the blood–brain barrier (BBB) and act as activators of the HPA axis. The activation of the HPA axis leads to an increased intestinal permeability, with further bacterial migration and additional alteration in the gut microbial composition.

It is of great importance that increased activity and dysregulation of the HPA axis are observed in human patients with mental disorders [162]. Increased cortisol levels are also associated with cognitive impairment, which can affect behavior [163]. Stressful situations, with further activation of the HPA axis, can also lead to changes in the gut microbiome. Altered microbiota composition has been observed following exposure to various stressors (including physical restraint, noise, or maternal separation) in animal models. Until now, various mechanisms have been proposed to clarify the link between the gut microbiota and HPA axis. As was mentioned above, gut dysbiosis may contribute to the enhanced production of pro-inflammatory cytokines. Some of these small bioactive molecules (including TNF-alpha and IL-6) might cross the blood–brain barrier (BBB) and act as activators of the HPA axis [164]. The activation of the HPA axis contributes to an increased intestinal permeability, which results in an alteration in the intestinal microbial composition and neurotransmitter production (including serotonin), as well as bacterial migration (Figure 2).

The HPA axis can be also activated by a bacterial endotoxin lipopolysaccharide (LPS), which stimulates systemic inflammation and translocates from the gut to the brain via the leaky mucosal barrier. Oral administration of *Escherichia coli*-derived LPS induced abnormal behavior and increased glucocorticoid receptor pathway genes in mice [165]. Similarly, *Escherichia coli* colonization in germ-free mice enhanced the HPA axis response to stressful situations [166]. Taken together, emerging evidence has indicated that changes in the gut microbiome can influence brain functions, which results in HPA axis dysregulation, chronic systemic inflammation, neurotransmitter imbalance, and finally, behavioral disorders.

The HPA axis is generally considered to be the main mechanism of the canine stress response, and plasma levels of cortisol have been widely used as measures of stress in dogs [167,168,169]. In one study, the authors focused on the comparison of the gut microbiota between dogs exhibiting aggressive, phobic, or normal behavior, with specific associations with adrenocortical activity. The obtained results revealed the specific gut microbiome composition of aggressive dogs, compared to phobic and normal individuals, with no differences in fecal cortisol levels [71]. These contradictory results (aggressive dogs are well known to have higher blood concentrations of cortisol) may be related to the methodology of the experiment since cortisol levels were tested in fecal samples, not within the blood. This necessitates further experimental study to deeply investigate the mechanisms underlying the relationship between the gut microbiome, the activity of the HPA axis, and canine behavioral disorders.

## 3. Fecal Microbiota Transplantation (FMT)

Accumulated data show that mammals’ gut microbiome, immune system, and nervous system are in continuous communication and influence animal physiology and behavior. According to human studies, the gut microbiome appears to be altered in people with depression disorders [54,55,56], anxiety [57,58,59], schizophrenia [60,61], posttraumatic stress disorder (PTSD) [62,63], or Alzheimer’s disease [70]. A different composition of the gut microbiota is also related with behavioral disorders in dogs, including aggressive and phobic behavioral disorders [71,72,73,74]. Thus, the modification of the gut microbiome can potentially be a helpful tool for treating mental health disorders. There are various strategies to manipulate gut microbiota, such as dietary changes, the administration of prebiotics, probiotics, or postbiotics, or fecal microbiome transplantation (FMT) [170,171,172]. The FMT method is based on transferring the fecal matter from a donor into the gastrointestinal tract of a recipient in order to modulate the gut microbiota. FMT is currently indicated for the treatment of debilitating gastrointestinal infections [173]. Preclinical and clinical data suggest that FMT is a promising strategy to meliorate psychiatric disorder symptoms [170]. Studies showed that transplanting the fecal microbiome from depressed humans to microbiota-depleted rats and mice can induce depressive-like and anxiety behaviors in recipients [174,175,176]. FMT was also used to correct intestinal flora and intestinal barrier damage in rats with stress-induced depressive-like behavior. Animals in this study were exposed to different stressors (including social isolation, heat stress, and restraint stress) for 4 weeks. Subsequently, the FMT procedure was performed by using fresh fecal samples from the control group. The obtained results revealed an improvement in depressive-like behavior, serotonin concentration, intestinal flora dysregulation, and the mucosal barrier in rats following FMT [177]. Similar studies were also performed on human patients with major depressive disorder. The FMT method was used as an add-on therapy and showed correction of patients’ depressive symptoms after 4 weeks following transplantation [178]. These beneficial therapeutic effects of FMT were also visible in patients with irritable bowel syndrome and co-occurring psychiatric symptoms (anxiety and depression behaviors) [179,180]. Although the research in this field is far from complete, the potential use of FMT treatment to alleviate anxiety and depression behaviors in human patients is promising.

FMT is also a recently adapted therapeutic approach in dogs; however, studies on its application are limited (Table 2).

In one study on dogs with acute hemorrhagic diarrhea syndrome, the FMT treatment did not have any clinical benefit [181]. On the other hand, a study in dogs with acute diarrhea treated with either fecal microbiota transplantation or metronidazole revealed beneficial effects of the FMT procedure. Dogs treated with metronidazole did not show proper microbial and metabolic profiles 28 days after, whereas the FMT treatment effectively stabilized microbiome parameters 7 days following the treatment [182]. Fecal transplantation has recently been tested as a treatment for canine IBD. The FMT procedure was performed in 16 dogs with an idiopathic IBD, unresponsive to common therapies. The results showed a clinical improvement in most of the patients after transplantation (by oral and endoscopic methods) [183]. Similarly, another study examining the FMT method as a treatment for canine IBD showed an improvement in clinical signs (including vomiting, diarrhea, and weight loss) after fecal implantation. According to the authors, the observed improvements were related to the changes in microbiota composition, especially the increase in *Fusobacterium* [184]. The effects of FMT as an adjunctive therapy were also evaluated in dogs with chronic enteropathies. The obtained results showed good clinical outcomes and suggest that FMT can be useful as an additive to standard therapy in dogs with chronic enteropathy [185]. Additionally, the FMT therapy has been recently recognized as a possible new therapeutic approach for canine atopic dermatitis [186].

Although the FMT procedure has been tested as a novel therapeutic approach targeting various canine somatic disorders, there is no study investigating the possible effect of this procedure on meliorating behavioral disorder symptoms.

## 4. Conclusions

Canine behavioral disorders have become one of the most common concerns and challenging issues among dog owners nowadays. It seems important to investigate the etiopathogenesis of canine mental disorders and look for more effective therapeutic agents or medical interventions to improve the mental health of dogs and, at the same time, the comfort of life of their caregivers.

Recently, scientific attention has been paid to the gut microbiota as a target in the treatment and prevention of abnormal behaviors. The exact mechanisms by which the gut microbiota modulates mental health are not fully understood. However, it is well known that the gut microbiome can influence the animal central nervous system via various mechanisms such as the vagus nerve, neurotransmitter level regulation (serotonin and dopamine, among others), production of metabolites (especially short-chain fatty acids), and the modulation of the HPA axis or inflammatory state within the organism. All of these factors sorely impact not only the somatic condition of the dog but also its mood and behavior.

However, currently, the influence of the gut microbiota on behavioral disorders is more recognized in human medicine compared to canine studies. The possible relationship between altered levels of crucial mental health neurotransmitters (such as serotonin, dopamine, and GABA), undesirable canine behaviors, and the condition of the gut microbiota should be examined. In particular, according to human and rodent studies, fecal microbiome transplantation could be a beneficial tool for treating mental health disorders also in canine patients.

In conclusion, future studies investigating the relationship between the brain–gut axis and canine behavioral disorders should incorporate a wider set of biomarkers, neurotransmitters, metabolites, and laboratory methods to test various interactions between the brain and the microbiome.

## Figures and Tables

**Table 2:** Summarized data on fecal microbiome transplantation (FMT) procedures performed in dogs with various diseases cited in the current review. The table shows studies using the FMT procedure as a treatment targeting various canine diseases, with the number of recipients enrolled in the study, the FMT method performed, and observed effects.

Fecal Microbiome Transplantation (FMT) Procedure in Dogs			
Recipient	No of Recipients	FMT Method	Effects
Dogs with acute hemorrhagic diarrhea syndrome [181]	8	Endoscopic	No clinical benefit; however, increased abundance of SCFA-producing bacteria (beneficial for the organism) was observed
Dogs with acute diarrhea [182]	11	Rectal enema	Fecal consistency significantly improved in all dogs, with proper microbial (based on dysbiosis index) and metabolic profiles (in contrast to dogs treated with metronidazole)
Dogs with inflammatory bowel diseases [183,184]	16 [183]; 9 [184]	Oral/endoscopic [183]; rectal enema [184]	Clinical improvement in most of dogs [183]; improvement in canine inflammatory bowel disease activity index in all dogs [184]
Dogs with chronic enteropathies (FMT used as add-on therapy) [185]	41	Rectal enema	Thirty-one dogs responded to treatment, resulting in improved fecal quality and/or activity level
Dogs with atopic dermatitis [186]	12	Oral	Eleven dogs presented significantly decreased skin lesions and pruritus scores and beneficially changed gut microbiota

## Data Availability

Data sharing is not applicable.
